# Novel *Siprulina platensis* Bilosomes for Combating UVB Induced Skin Damage

**DOI:** 10.3390/ph16010036

**Published:** 2022-12-27

**Authors:** Mariam Zewail, Passent M. E. Gaafar, Nancy Abdel Hamid Abou Youssef, Merhan E. Ali, Mai F. Ragab, Miranda F. Kamal, Mohamed H. Noureldin, Haidy Abbas

**Affiliations:** 1 Pharmaceutics Department, Faculty of Pharmacy, Damanhour University, Damanhour P.O. Box 22511, Egypt; 2 Department of Pharmaceutics, Division of Pharmaceutical Sciences, College of Pharmacy, Arab Academy for Science, Technology and Maritime Transport, Alexandria P.O. Box 1029, Egypt; 3 Department of Pharmaceutics, Faculty of Pharmacy, Pharos University in Alexandria (PUA), Alexandria P.O. Box 21500, Egypt; 4 Department of Pathology, Faculty of Veterinary Medicine, Cairo University, Giza P.O. Box 12211, Egypt; 5 Pharmacology Department, School of Life and Medical Sciences, University of Hertfordshire Hosted by Global Academic Foundation, Cairo P.O. Box 11835, Egypt; 6 Pharmaceutical Analytical Chemistry Department, Faculty of Pharmacy, Damanhour University, Damanhour P.O. Box 22511, Egypt; 7 Department of Biochemistry, Division of Clinical and Biological Sciences, College of Pharmacy, Arab Academy for Science, Technology and Maritime Transport, Alexandria P.O. Box 1029, Egypt

**Keywords:** bilosome, Spirulina, antiaging, ultraviolet B skin damage, skin delivery

## Abstract

The recent interest in bioactive compounds from natural sources has led to the evolution of the skin care industry. Efforts to develop biologically active ingredients from natural sources have resulted in the emergence of enhanced skin care products. Spirulina (SPR), a nutritionally enriched cyanobacteria-type microalga, is rich in nutrients and phytochemicals. SPR possesses antioxidant, immunomodulatory, and anti-inflammatory activities. Spirulina-loaded bilosomes (SPR-BS), a novel antiaging drug delivery system, were designed for the first time by incorporation in a lecithin–bile salt-integrated system for bypassing skin delivery obstacles. The optimized BS had good entrapment efficiency, small particle size, optimal zeta potential, and sustained drug release pattern. Blank and SPR-loaded BS formulations were safe, with a primary irritancy index of <2 based on the Draize test. In vivo tests were conducted, and photoprotective antiaging effects were evaluated visually and biochemically by analyzing antioxidant, anti-inflammatory, and anti-wrinkling markers following ultraviolet (UV) B irradiation. Results of biochemical marker analysis and histopathological examination confirmed the superior antiaging effect of SPR-BS compared with SPR. Thus, SPR-loaded BS is a promising nanoplatform for SPR delivery, can be used for treating UV-induced skin damage, and offers maximum therapeutic outcomes.

## 1. Introduction

The skin is an important barrier organ that prevents dehydration and shields against the external environment. Being the largest organ in the body, it is highly exposed to the outer environment, suffering both intrinsic and extrinsic aging. Intrinsic or chronological skin aging encompasses irreversible natural aging with time or due to hereditary factors, whereas extrinsic skin aging predominantly results from continuous exposure to heat, smoking, pollutants, ultraviolet radiation (UVR) (photoaging), and stress as well as an unhealthy lifestyle [1,2]. In age-related skin changes, fine wrinkles, atrophy, and trans epidermal water loss might be realized as the cumulative effect of intrinsic factors, whereas exposure to external environmental factors often results in coarse wrinkles, skin dryness, loss of elasticity, laxity, and appearance of rough-texture due to the reduction of collagen, elastin fibers, and hyaluronic acid [1,3].

UV radiation has pros and cons on the skin and body condition. Phototherapy has a great impact on the treatment outcomes of several diseases, including skin diseases [4]. However, prolonged exposure to direct sunlight and UV rays, particularly UVB radiation with a wavelength of 280–320 nm, can initiate reactive oxygen species (ROS) generation through cellular oxidative metabolism, resulting in lipid, protein, and DNA damage. Also, the level of antioxidants in the skin may be reduced [5]. Furthermore, prolonged UV exposure may lead to collagen degradation as a result of matrix metalloproteinase (MMPs) activation [6].

Youth and beauty are highly valued in society. New products that enhance both the health and beauty of the skin are continuously developed. Bioactive ingredients, for instance, vitamins, phytochemicals, enzymes, antioxidants, essential oils, and extracts are added to antiaging cosmetic products [7]. Natural source ingredients are increasingly preferred due to their numerous beneficial effects compared with their synthetic counterparts. The natural cosmetics industry remains a smaller fraction of the market compared with traditional cosmetics, although, they are mild, biodegradable, safe, and have lesser side effects. Oceans are a valuable source of unique compounds with industrial application potential including cosmetic ingredients. Among these, bacteria and algae constitute a major source of active ingredients [8]. Algae are classified based on their morphological features into microalgae (i.e., unicellular, such as diatoms, or multicellular) or macroalgae (sometimes referred to as seaweeds). Marine algae are classified into major distinct classes based on the presence of specific pigments. For instance, algae can be brown, blue-green, green, or red [7,9].

Cyanobacteria, known as blue-green algae, are prokaryotic microalgae, whereas eukaryotic microalgae include diatoms and green algae [10]. Blue-green algae contain many effective ingredients. Based on the market intelligence agency Mintel, blue-green algae showed the highest skincare trends in 2019 [11]. Cyanobacteria can retain water and produce UV protective compounds, allowing them to be used as natural UV filters and moisturizers. They are also a rich source of antioxidants that neutralize free radicals [11]. In addition, they are a crucial source of exocellular polysaccharides that are used as stabilizers in the food industry as well as hydrating agents in cosmetics and pharmaceuticals [10].

Few reports exist on the use of cyanobacteria in cosmetic applications. Some molecules from different cyanobacteria species had promising results in skin care, such as the antioxidant effect of the methanolic extract of exopolysaccharides from *Arthrospira platensis* [12]. Previous clinical and preclinical trials found that Spirulina (SPR) has antioxidant, immunomodulatory, and anti-inflammatory effects. These effects can counteract the effect of ROS as SPR can prevent and inhibit lipid peroxidation, scavenge free radicals, or increase superoxide dismutase (SOD) and catalase (CAT) activities [13,14]. SPR is a unicellular cyanobacterial-type microalga that grows at pH 10–12. SPR was safely used in Africa for several years [15]. SPR is a nutritionally enriched algae that have been globally cultivated as a dietary supplement, owing to its versatile biological and nutritional profile [16]. It is highly rich in nutrients, phytochemicals, proteins, and carbohydrates [17]. Among these various proteins are phycobiliproteins, namely phycocyanin, allophycocyanin, and phycoerythrin, which represent nearly 60% of its dry weight. Phycocyanin possesses many beneficial effects as a nutraceutical, as well as a therapeutic agent with strong anticancer, antidiabetic, hepatoprotective, and neuroprotective effects due to its strong antioxidant activity. It scavenges free radicals and protects against lipid peroxidation [18,19]. In addition, it reduces inflammation and allergies [20]. SPR is rich in fatty acids. The major lipids found in SPR are γ-linolenic acid and palmitic acid. Furthermore, SPR is a rich source of many minerals and vitamins including vitamin B, calcium, potassium, magnesium, selenium, iron, and zinc [21]. Also, SPR is rich in carotenoids and chlorophyll [22].

Studies have confirmed that carotenoids and chlorophyll can be used to combat skin aging and protect the skin against UV-induced photo-oxidation [14,23]. Thus, SPR has several pharmacological activities, such as antimicrobial, antioxidant, antihypertensive, anti-inflammatory, immunomodulatory, and anticancerogenic properties [16,24]. These attributes make SPR an attractive ingredient, especially for the formulation of green and ecofriendly cosmetics [15]. Based on this, recent trends use SPR food supplements as “nutricosmetics” that not only help to prevent diseases and promote health, but also boost the natural beauty of the skin, nails, and hair [25,26]. Therefore, the need for developing a novel topically-applied, a natural compound that could offer high UVR photoprotective properties with minimal side effects has emerged. Nevertheless, the major limitations of current traditional topical cosmeceutical therapy are limited skin penetration and contact time due to the stratum corneum barrier [27].

Nanotechnology can provide a novel method to overcome these obstacles. Nanotechnology can offer remarkable and innovative solutions for product delivery. The small particle size and high surface area of nanoparticles increase contact time and the penetration of active molecules into the skin. In addition, they have high stability, site-targeted drug delivery, controlled and sustained drug release, and reduced side effects [11,28]. Nanosystems such as liposomes, ethosomes, transferosomes, and bilosomes (BS) have shown promising results in dermal drug delivery [29]. Nonetheless, despite the interest of the cosmetic market in naturally derived skin care products, the promising potential of marine-derived natural products as a source for active compounds with antiaging effects has remained relatively undiscovered until recently [12,30].

Heretofore, a few studies have aimed to study and demonstrate the effect of a nanoplatform incorporating SPR. To our knowledge, no reports of SPR nanoformulations’ effects on ultraviolet B-mediated skin aging. Thus, the aim of this study is to develop a novel topically-applied antiaging nanodelivery system encapsulating SPR, followed by in vitro and in vivo assessment to confirm the superior efficacy of this novel nanoplatform.

## 2. Results and Discussion

### 2.1. Physicochemical Characterization of Blank BS and SRP-BS

This study investigated the use of a SPR nanovesicular system to enhance the penetration and deposition of SPR in skin tissue as a protective topical antiaging platform. BS are bile salt-stabilized nanovesicular carriers that were first described by Conacher et al. [31]. Integrating naturally occurring lipids with bile salts leads to the formation of a self-assembling structure called the “bilosome” (BS). Bile salts are endogenous biosurfactants with unique chemical properties used as absorption enhancers [32], capable of enhancing the transportation of bioactives across biological barriers through membrane destabilizing activity, lipid fluidization, and interaction with dermal collagen and keratin filaments. Thus, the dual permeation enhancing effect of both phospholipids and bile salts can overcome the skin barrier with superior drug deposition in skin layers and minimal systemic absorption, leading to reduced side effects [33]. Thin-film hydration technique was adopted to prepare different formulations of BS, with PBS as the hydrating medium PBS (pH 7.4). SDC was selected as a bile salt in BS preparation based on its previously reported permeation enhancing effects in other transdermal preparations [33,34,35,36]. In this study, a crude SPR extract was used rather than a pure extract of separate active constituents because SPR contains several nutrients that exhibit pharmacological activity [37].

Salem et al. [38] reported that BS prepared using sodium cholate or sodium taurocholate showed lower EE% than SDC. SDC increases bilayer membrane elasticity and drug solubility in the membrane due to its surface-active property and ability to integrate into the surface of the bilayer membrane, thus increasing EE% [38].

Different ratios of PC to SDC were studied (10:1% and 4:1% *w*/*w*) to optimize the BS formulation with respect to PS, ZP, and polydispersity index. The quality attributes of blank BS and SPR-BS are shown in Table 1. Particle size and zeta potential distribution are illustrated in Figures S1 and S2, respectively.

ZP value ±30 mV is considered optimum and confers stability to the prepared nanocarriers’ dispersion [39,40]. ZP values of the prepared bilosomal formulations ranged from −28.4 ± 1.6 to −34.5 ± 1.3 mV; indicating the physical stability of the prepared bilosomal dispersion. All formulations carried negative surface charges as a result of SDC incorporation in their formulation [41]. Regarding bile salt concentration, upon increasing SDC concentration, zeta potential increased (Table 1). The integration of the anionic SDC with phospholipid vesicle membranes increased net negative charge, with high repulsive force between vesicles. These results agree with the previously reported data by Abbas et al. [36], Abdelalim et al. [32], and El Naggar et al. [42].

**Figure 1:**
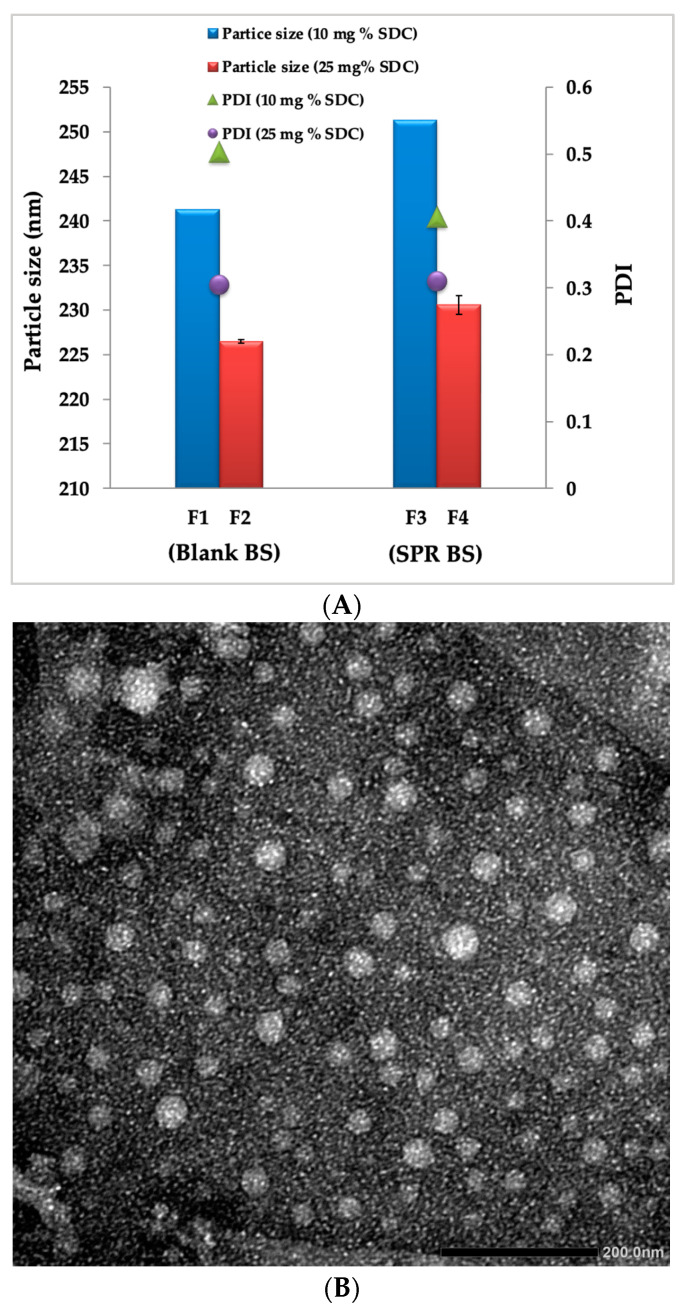
(**A**) Effect of SDC concentration and drug loading on particle size and PDI of BS and (**B**) TEM micrograph of selected SPR-BS formulation (F4).

Particle size influences vesicle properties, and smaller vesicles show more drug localization than larger ones. In this study, particle size ranged from 226.5 ± 1.65 to 251.3 ± 1.21 nm. In fact, the recorded size range of the prepared SPR-BS (Table 1) appears favorable for the desired objective, as it was reported that this particle size range possesses a higher UV-blocking effect, and consequently, better skin protection can be attained [43]. A size range smaller than 300 nm guarantees more drug localization within the skin layers, with the ability to deliver the contents into the deep layers of the skin [44,45]. Notably, increasing the SDC ratio caused a significant decrease in particle size (Table 1, Figure 1A). This may be attributed to the effect of SDC on decreasing surface tension and the interfacial tension between vesicle bilayers, which increased vesicle membrane flexibility [46], and consequently, the space among vesicle bilayers [38].

Particle dispersion with PDI values from 0 to 0.4 are considered homogenous [38]. In our study, PDI values ranged from 0.30 to 0.50 (Table 1), which indicates limited size distribution and good uniformity. Moreover, SPR loading significantly affected the characteristics of BS. PS of SPR-BS increased significantly compared to blank BS upon loading BS with SPR (Student’s *t* test; *p* < 0.05) (Figure 1A). PS values of F1 and F3, which both contain 10% SDC, were 241.3 ± 1.87 and 251.3 ± 1.2, respectively. By contrast, PS values of F2 and F4, which both contain 25% SDC, were 226.5 ± 1.65 and 230.6 ± 1.3, respectively. These findings are consistent with previously reported results [47,48].

### 2.2. Percentage Entrapment Efficiency

Encapsulating significant quantities of SPR is a meaningful character in the evaluation of a nanoformulation as it reflects its capability in retaining bioactive compounds in the bilayer membrane or the central core of the nanodelivery system. The amount of entrapped SPR was determined using the spectrophotometric method. SPR-BS yielded a high entrapment efficiency percentage (EE%) of 89.5 ± 1.08 and 85.2 ± 1.5 for F4 and F3, respectively (Table 1). This may be attributed to the surface-active properties of SDC, as it might integrate into the lipid bilayer membrane enhancing the solubility of SPR. Furthermore, the incorporation of the hydrophobic SDC in the BS structure resulted in an effective intermolecular association of the hydrophobic drug SPR inside the hydrophobic tails of the phospholipid bilayer, producing high EE% [36]. Consistently, El-Nabarawi et al. [33] reported that BS prepared with SDC had the highest EE% value compared with other formulations prepared using other types of bile salts of higher HLB values [33]. Increasing the concentration of bile salts from 10 mg% to 25 mg% (in F3 and F4, respectively) significantly increased EE% from 85.2 ± 1.5 to 89.5 ± 1.08 (Table 1). Consistently, Zafar et al. [49] reported that increasing SDC concentration had a synergistic effect on EE% because SDC has surfactant properties and its incorporation into the lipid membrane surface increases membrane flexibility and drug solubility in the lipid membrane, and hence EE% [49].

### 2.3. Transmission Electron Microscopy

In order to examine the morphology of the selected SPR-BS formulation (F4), TEM was employed. TEM micrographs revealed characteristic well-dispersed unilamellar nanovesicles with uniform spherical shapes with no aggregation shell-core vesicles (Figure 1B). The particle size of the TEM micrograph is along with our previously mentioned results in Section 2.1.

### 2.4. In Vitro Release Studies

**Figure 2:**
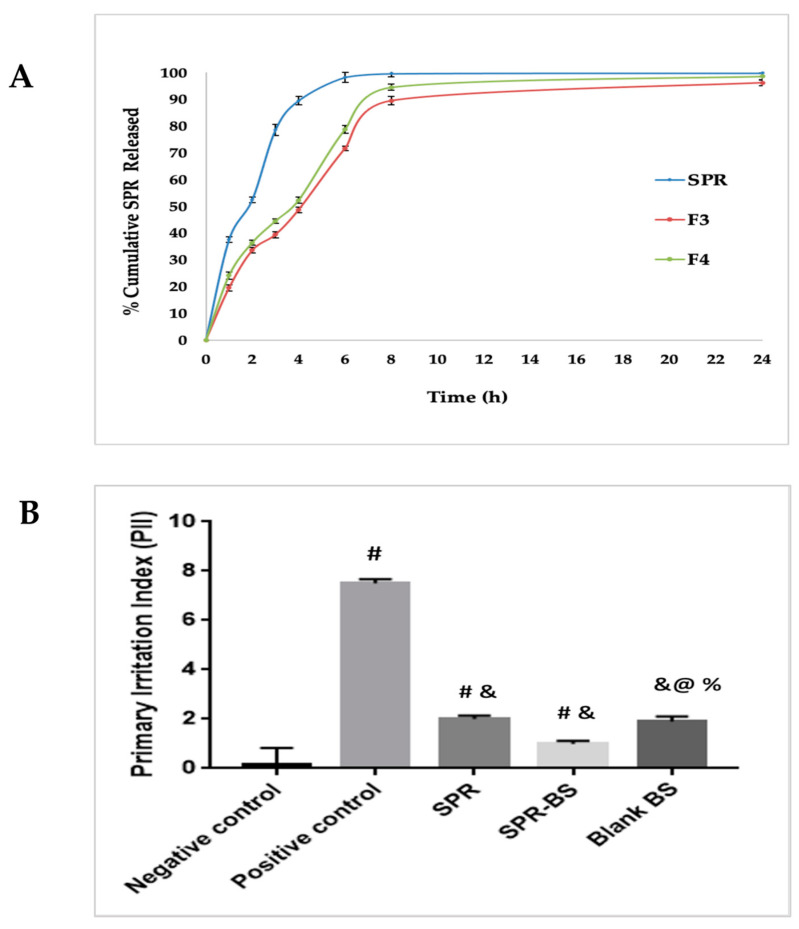
(**A**) Cumulative percentage SPR released from SPR and SPR BS. (**B**) Primary irritation index of different experimental groups in Draize method. Values are represented as mean ± SD. # significant from negative control group. & Significant from positive control group. % Significant difference from blank BS. @ significant from SPR group. Significant difference was conducted at *p* < 0.0001.

The effectiveness of a drug delivery system can be governed by the release behavior of the active substance. Figure 2A shows the cumulative release profiles of the SPR solution, F3 and F4 in PBS (pH 6.8). SPR UV spectrum and calibration curve in PBS pH 7.4 are illustrated in Figure S3A,B. Both F3 and F4 BS formulations demonstrated a biphasic release pattern with gradual release of SPR up to 39.4% and 36.4% in 3 h, respectively. The sustained release profile of SPR from BS may be attributed to the effect of BS in controlling drug release. These findings are consistent with the results reported by Abbas et al. [36]. F4 offered a faster release rate than F3—94.5% compared with 89.5% after 8 h, respectively (Figure 2A). The faster release of F4 than F3 may be because F4 contains a higher amount of SDC, which can act as a permeation enhancer when included in the BS structure, where its incorporation in the phospholipid bilayer resulted in the liberation of the loaded lipophilic drug [35]. Moreover, SDC may combine with vesicle bilayers leading to their conversion into mixed micelles, which in turn can increase drug solubility and enhance drug release. Similar findings were reported by Albash et al. [50]. Consistently, Khalil et al. [35] reported that the percentage of tizanidine hydrochloride released after 8 h from BS increased upon increasing SDC from 5 to 20 mg [35].

Statistical analysis of release results by one-way ANOVA (*p* < 0.001) revealed a significant difference in BS formulation and SPR suspension and a significant difference in release profiles between F3 and F4. Release data were fitted to zero-order, first-order, and Higuchi and Korsmeyer-Peppas equation (Table 2). SPR release from suspension, F3, and F4 fitted the first-order release model because it had the highest R^2^ values. First-order release kinetics describes the release of a drug entrapped within a porous membrane by diffusion [48].

### 2.5. Effect of Storage

Testing the stability of the prepared BS during storage for 3 months at 4 °C revealed that all the analyzed parameters (PS, PDI, ZP, and &EE) of both bilosomal formulations (F3 and F4) remained constant during the storage period. It should be noted that both formulations did not show any reduction of EE on storage when referred to the fresh formula by student *t*-test (*p* < 0.05), thus, highlighting the actual high stability of the developed vesicular formulations. This may be attributed to the high negative charge due to the anionic nature of SDC [51].

Conclusively, from all the in vitro results, F4 was selected for in vivo studies since it had a smaller particle size (230.6 ± 1.3), higher entrapment efficiency (89.5 ± 1.08) suitable zeta potential (−34.5 ± 1.3), and demonstrated stability during storage.

### 2.6. In Vivo Studies

The choice of 1.125% SPR was based on Gunes et al. [37], who reported that skin cream containing 1.125% SPR crude extract had the maximum proliferative effect on skin cells and supported their findings by immunohistochemical assays and cell culture results [37].

#### 2.6.1. Skin Irritancy Test

As shown in Figure 2B, following visual examination, the recorded scores for the SPR suspension (2.3 ± 0.12), blank BS (1.8 ± 0.16), and selected SPR-BS formulation (F4) (1 ± 0.13) revealed significantly lower dermal irritation than the positive control group (7.5 ± 0.16) (*p* < 0.0001). Thus, the minimal erythema shown by SPR-BS confirms the nonirritancy of the developed formulation.

#### 2.6.2. UVB Exposure Test

**Figure 3:**
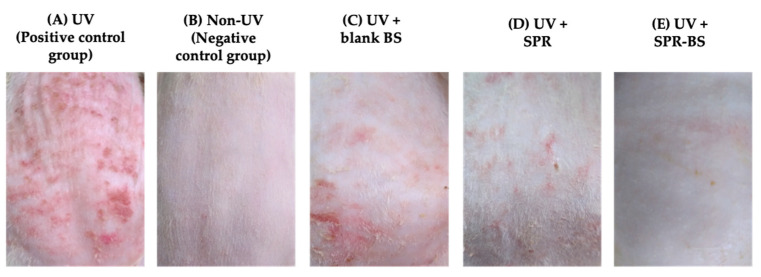
Photographs of the dorsal rats’ skin (**A**) UVB irradiated group, (**B**) Non UVB irradiated group, (**C**) irradiated and topically pretreated with blank BS, (**D**) irradiated and topically pretreated with SPR and (**E**) irradiated and topically pretreated with SPR-BS.

The effect of topical pre application of SPR suspension, SPR-BS, and blank BS on UV-induced photo aging was studied by visual examination of the dorsal mouse skin. The non-UV-treated group demonstrated intact skin, with a smooth surface (Figure 3). By contrast, edema, erythema with thick skin, and notable scars were observed after irradiation in the positive control group (Figure 3A). The observed skin thickening could be due to epidermal hyperplasia and thickening of the stratum corneum [52]. In the negative control group, none of these signs were observed (Figure 3B). The pre application of blank BS showed prominent protective effects compared with the positive control group. This innate bioactivity may be attributed to the curative effect of phospholipids, as they can replenish and regain skin barrier function [36].

By contrast, after the pre application of SPR-BS, the skin appeared intact and free from any signs of wrinkling and showed better improvement compared with treatment with SPR suspension (Figure 3D,E). This is expected because the penetration enhancing abilities of BS overcome the stratum corneum barrier. Thus, following topical application, the loaded drug efficiently penetrated intact skin to deeper skin layers where it offered a promising protective antiaging effect. In addition, several reports [33,34,36] have confirmed the innate capability of bile salts in BS to interact with skin keratocytes. This adds to the effect of SPR in protecting the skin from photo damage [15]. Thus, SPR-BS is an efficient topical antiaging tool.

#### 2.6.3. Biochemical Analysis

##### Anti-Inflammatory Markers

The repair of tissue damage may occur by the inhibition of the inflammatory cascade. Topical applications of antioxidant and anti-inflammatory products can correct impaired skin conditions, by replenishing the natural balance and functionality of the skin. Nanocarriers have been utilized to achieve this goal, because nanocarriers, including liposomes and BS, are considered effective systems for topical and transdermal drug delivery [53,54]. Proinflammatory cytokines, such as IL-6 and TNF-α, are released in response to UVB-induced skin damage [55,56]. Moreover, the level of proinflammatory cytokines released is related to the severity of UVB-induced skin damage and chronic inflammation [57,58].

**Figure 4:**
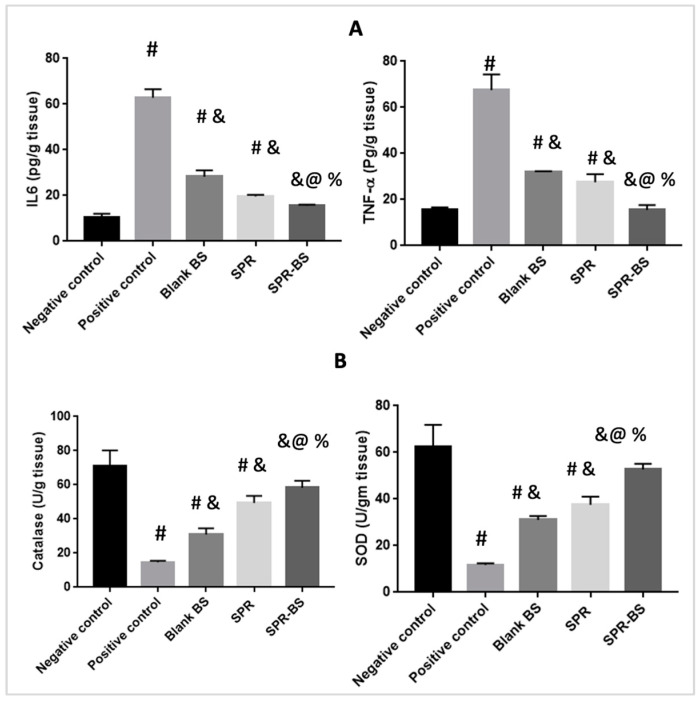
Level of different biomarkers at the end of the experiment. (**A**) Level of anti-inflammatory parameters and (**B**) level of antioxidant parameters. Values are represented as mean ± SD. # significant from negative control group. & Significant from positive control group. % Significant difference from blank BS. @ significant from SPR suspension. Significant difference was conducted at *p* < 0.0001.

Levels of IL-6 and TNF-α were assessed at the end of the experiment (Figure 4A). The positive control group showed the highest level of IL-6 and TNF-α. By contrast, the negative control group showed the lowest levels of inflammatory mediators, followed by groups treated with SPR-BS, SPR, and blank BS. Levels of IL-6 and TNF-α in the positive control group were higher than that in the negative control group by 6- and 4.3-fold, respectively. TNF-α levels were 15.5, 31.95, and 27.5 pg/g tissue in groups treated with SPR-BS, blank BS, and SPR suspension, respectively. Surprisingly, the group treated with blank BS showed a significant reduction in both IL-6 and TNF-α levels by 2.2- and 2.1-fold, respectively compared with the positive control group. This may be attributed to the shielding and protective effects of the nanovesicles [59]. The SPR suspension-treated group demonstrated a reduction in IL-6 and TNF-α by 3- and 2.4-fold, respectively, compared with the positive control group. Consistently, Abbas et al. [36] reported that blank BS provides protection against UVR. SPR-BS decreased TNF-α and IL-6 levels by 4.3- and 4-fold, respectively, compared with the positive control. Moreover, SPR-BS decreased TNF-α and IL-6 levels by 2- and 1.8-fold, respectively, compared with the SPR-treated group, demonstrating the superiority of SPR-BS over SPR suspension.

##### Antioxidant Markers

ROS are reactive metabolites that are formed after exposure to UV-A and UV-B radiation. They are considered a part of normal cell regulation and are influenced and regulated by cellular antioxidants. However, increased ROS levels and reduced cellular redox homeostasis promote skin carcinogenesis and photoaging [60]. The antioxidant potential of SPR-BS was assessed by measuring SOD and catalase levels. SOD acts as the primary antioxidant enzyme that converts the superoxide radical into hydrogen peroxide and oxygen [61]. Oxidative stress occurs when there is an imbalance between free radical production and antioxidant levels. This was observed in the reduced activity of SOD. Furthermore, SOD is a major antioxidant in human fibroblasts and a causative factor in oxidative stress on telomere shortening [62]. Thus, enhancement of SOD activity can improve the prevention of oxidative stress and photo-induced aging of the skin.

Figure 4B shows the effect of different treatments on the level of antioxidant enzymes (SOD and catalase). Exposure to stress, such as UVR, reduces SOD and catalase levels [63]. Our results confirmed that the positive control group demonstrated a statistically significant reduction from the non-UVB irradiated control group by 4.8- and 5.3-fold for catalase and SOD, respectively. This is in good agreement with the data of Raza et al. [64] who reported that UVR increased lipid peroxidation and free radical production and recorded a significant change in the levels of antioxidant markers [64].

The negative control group showed the highest levels of catalase and SOD followed by the SPR-BS, SPR suspension, and blank BS groups. The positive control group had the lowest level of antioxidant markers (Figure 4B). Alternatively, significant changes in the level of antioxidant markers compared with the positive control was observed after topical administration of SPR suspension confirming the antioxidant effect of SPR. Further, an improvement in the levels of catalase and SOD in the SPR suspension group was demonstrated compared to the positive control group by 3.3- and 3.2-fold, respectively. This may be attributed to the ability of SPR to inhibit ROS-induced dermis damage [15]. Studies by Jozsa et al. [65] reported that the antioxidant effect of SPR extract was ~68% that of acsorbic acid [65]. Abdel-Moneim et al. [66] reported that the SPR extract could significantly scavenge ABTS and DPPH radicals in a dose-dependent manner [66]. SPR-BS could increase catalase and SOD levels by 4.5- and 3.9-fold, respectively, compared with the positive control group. SPR-BS appears to have superior antioxidant effects compared with the SPR suspension. This may be attributed to the effect of nanoencapsulation on improving drug penetration through the skin, and hence, better pharmacological outcomes can be attained [36]. The group treated with blank BS showed statistically significant differences in the levels of the antioxidant markers compared with the positive control, SPR suspension, and SPR-BS groups. Catalase levels in the blank BS and SPR suspension groups were 30.95 and 49.4 U/g tissue, respectively. By contrast, SOD levels in blank BS and SPR suspension groups were 31.05 and 37.5 U/g tissue, respectively. These findings confirm the shielding and protective effect of nanocarriers [36].

##### Antiwrinkling Markers

The clinical signs of skin aging, such as wrinkles and laxity, are mainly due to the cleavage of interstitial collagen and alterations in the extracellular matrix. UVB exposure stimulates free radicals’ production, which increase the production of MMPs, thereby increasing collagen and elastin degradation [67,68] and, eventually, wrinkle formation. UVB radiation stimulates keratinocytes to secrete IL-1, which activates the secretion of GM-CSF, triggering fibroblasts to increase neprilysin expression, thereby leading to the deterioration of the three-dimensional fibrous network of the skin; consequently, skin elasticity is reduced, and wrinkles appear [69,70].

**Figure 5:**
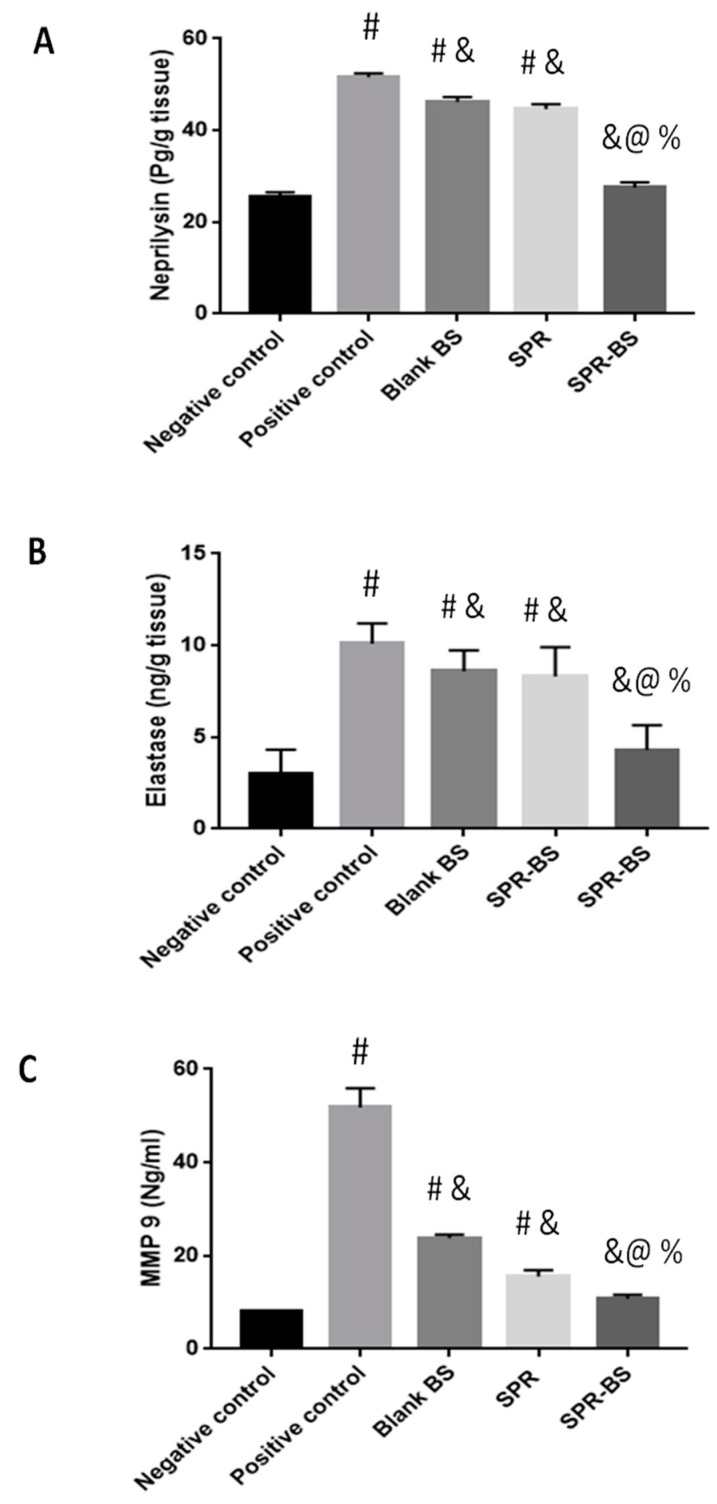
Level of anti-wrinkling parameters at the end of the experiment. (**A**) Neprilysin level, (**B**) elastase level and (**C**) MMP 9 level. Values are represented as mean ± SD. # significant from negative control group. & Significant from positive control group. % Significant difference from blank BS. @ significant from SPR suspension. Significant difference was conducted at *p* < 0.0001.

The levels of elastase, neprylysin, and MMP9 were evaluated at the end of the experiment. In the positive control group, the levels of elastase, neprylysin, and MMP9 increased by 3.3-, 2-, and 6-fold, respectively, compared with the negative control group (Figure 5). The negative control group recorded the lowest level of antiwrinkling parameters, followed by groups treated with SPR-BS, SPR suspension, and blank BS. Moreover, the negative control group showed statistically significant differences (*p* < 0.001) from all groups except the group treated with SPR-BS. Elastase levels were 3.3, 10.1, 8.6, 8.3, and 4.3 ng/g tissue in the negative control group; positive control group; and groups treated with blank BS, SPR suspension, and SPR-BS, respectively; neprilysin levels were 25.6, 51.6, 46.6, 44.6, and 27.5 ng/g tissue, respectively. Notably, groups treated with blank BS, SPR suspension, and SPR-BS were able to reduce MMP9 levels by 2.1, 3.3, and 4.97 folds, respectively compared with the positive control group.

Overall, a noticeable effect of SPR-BS compared to the drug suspension (*p* < 0.001) was observed and confirmed by visual inspection of the dorsal rat skin (Figure 3). Our findings illustrated the superiority of SPR-BS over SPR suspension and blank BS in improving the levels of anti-inflammatory, antioxidant, and antiwrinkling markers. Statistical analysis of results for all biochemical markers revealed no statistically significant difference in biomarker levels between SPR-BS-treated and negative control groups (*p* < 0.0001).

Our results are consistent with those of others [36,63,71] who have shown the superiority of nanosystems compared with drug suspension systems in combating UV-induced skin damage using herbal drugs, such as resveratrol [63,71] and luteolin [36]. This may be attributed to two reasons. First, BS promotes cutaneous drug penetration, and hence, enhances the pharmacological effects of the drug. The mechanism of enhanced cutaneous delivery of BS has been previously reported [33,72,73]. The enhanced delivery is mainly attributed to the effect of bile salts in improving vesicle deposition in the skin through micellar entrapment and solubilization of intercellular lipids. BS can enhance skin penetration by affecting protein denaturation and extraction in addition to enzyme inactivation. Moreover, bile salts can act as edge activators affecting membrane flexibility and altering the horny layer of the skin, thus facilitating vesicle penetration through the intact layers [33,72].

Second, the innate properties of SPR are enhanced by nanoencapsulation. SPR can act as an antioxidant, antiaging, antiwrinkling agent, in addition to being a sunscreen [15]. SPR was reported to reduce skin hyperpigmentation, protect against photoaging, and inhibit free radical production [74]. These effects of SPR may be attributed to its composition, because SPR contains several nutrients and ingredients. For example, SPR has two important phycobiliproteins: phycocyanin and allophycocyanin. Studies have reported that phycocyanin possesses antioxidant and anti-inflammatory properties [37]. Phycocyanin can inhibit proinflammatory cytokine formation, such as TNF, suppress cyclooxygenase-2 expression, and decrease prostaglandin E2 production [75]. Moreover, SPR contains carotene, which exhibits potent antioxidant and anti-inflammatory activities. Carotene was reported to protect cells against singlet oxygen-mediated lipid peroxidation [75].

In a clinical study conducted using 44 healthy volunteers, Souza et al. [74] reported that the addition of antioxidants, such as SPR, to the sunscreen formulation, resulted in a significant improvement in skin pigmentation and collagen degradation, and skin elasticity recovered 84d after treatment [74]. Moreover, Delsin et al. [76] reported that the gel cream formulated from SPR extract amended skin conditions and improved skin barrier and oil control function along with maintaining skin hydration [76].

### 2.7. Western Blotting

**Figure 6:**
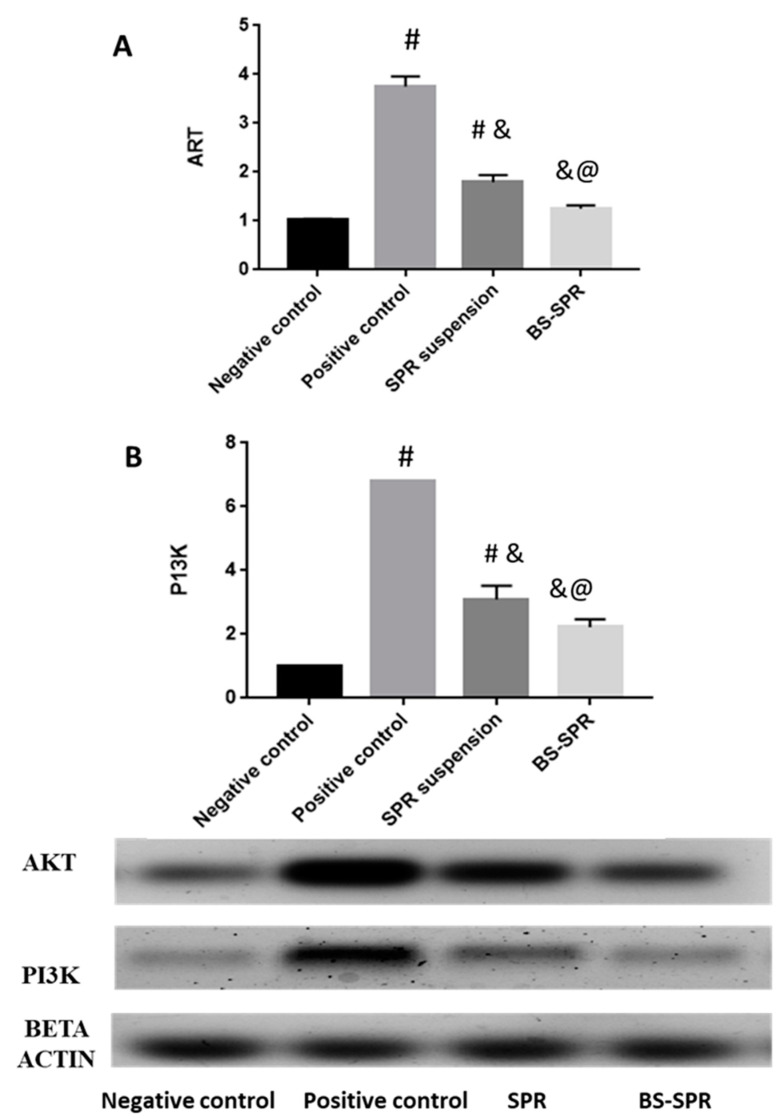
Effect of SPR suspension and SPR BS on (**A**) AKT and (**B**) PI3K level in skin. Values are represented as mean ± SD. # significant from negative control group. & Significant from positive control group. @ Significant from SPR suspension. Significant difference was conducted at *p* < 0.0001.

The phosphatidylinositol 3-kinase (PI3K)/protein kinase B (Akt) signaling pathway can control cell proliferation, differentiation, and migration; angiogenesis; and metabolism. Moreover, the PI3K/Akt pathway can mediate skin development and homeostasis [77]. We evaluated the effect of SPR and SPR-BS on AKT and PI3K levels (Figure 6). Analysis of protein levels revealed that the positive control group showed the highest expression of AKT and PI3K (Figure 6). Levels of AKT and PI3K were statistically significantly lower in SPR, and SPR-BS groups compared with the positive control group. The group treated with SPR-BS demonstrated no statistically significant difference from the negative control group. Results of western blotting revealed the ability of SPR to lower PI3K and AKT levels. Downregulation of PI3K is associated with decreased production of ROS, which is directly associated with skin aging [78].

### 2.8. Histopathological Analysis and Immunohistochemical Staining

**Figure 7:**
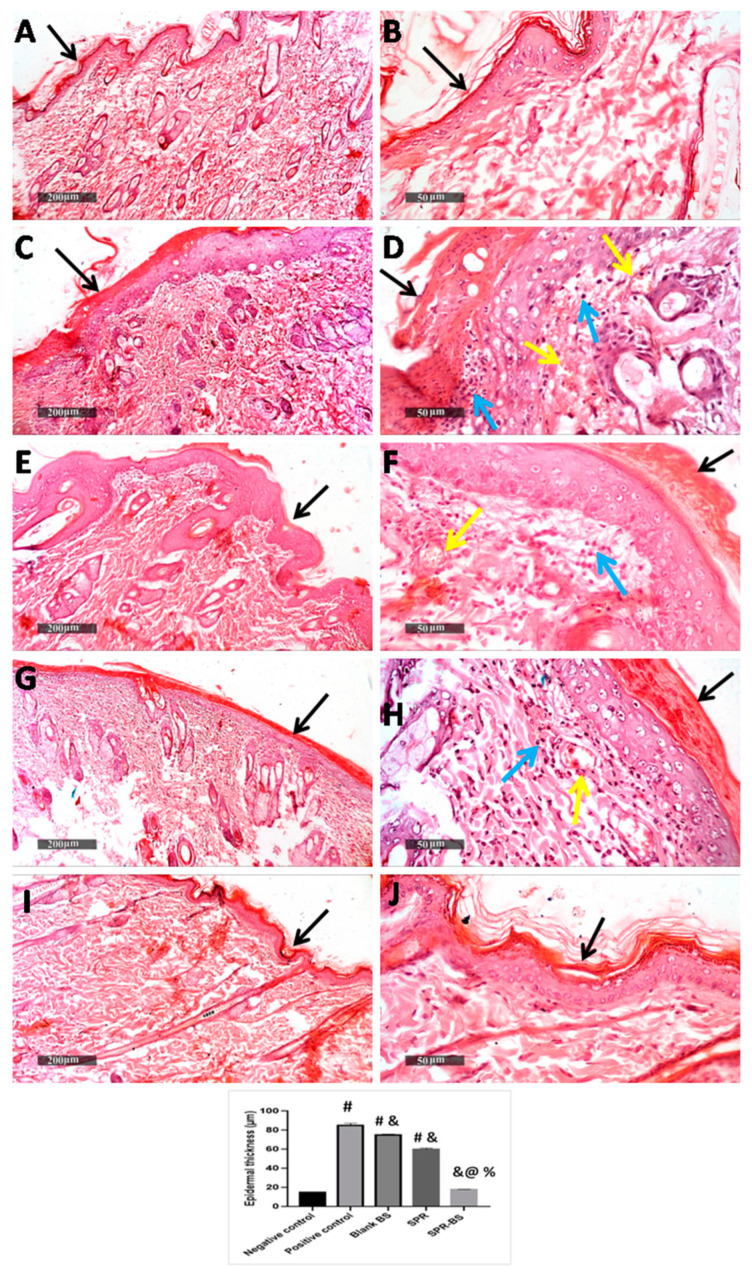
Photomicrograph of skin samples from UVB irradiated skin rats. (**A**,**B**) showing normal histological features of non-UV irradiated control group, (**C**,**D**) for UVB irradiated group, (**E**,**F**) for Blank BS treated group, (**G**,**H**) for SPR suspension treated group and (**I**,**J**) for SPR-BS treated group. (**H**,**E**) stain. Epidermis (black arrows), inflammatory cells (blue arrows), Congested BVs (yellow arrows). Values are expressed as mean ± SD. # significant from negative control group. & Significant from positive control group. % Significant difference from blank BS. @ significant from SPR suspension. Significant difference was conducted at *p* < 0.0001.

**Figure 8:**
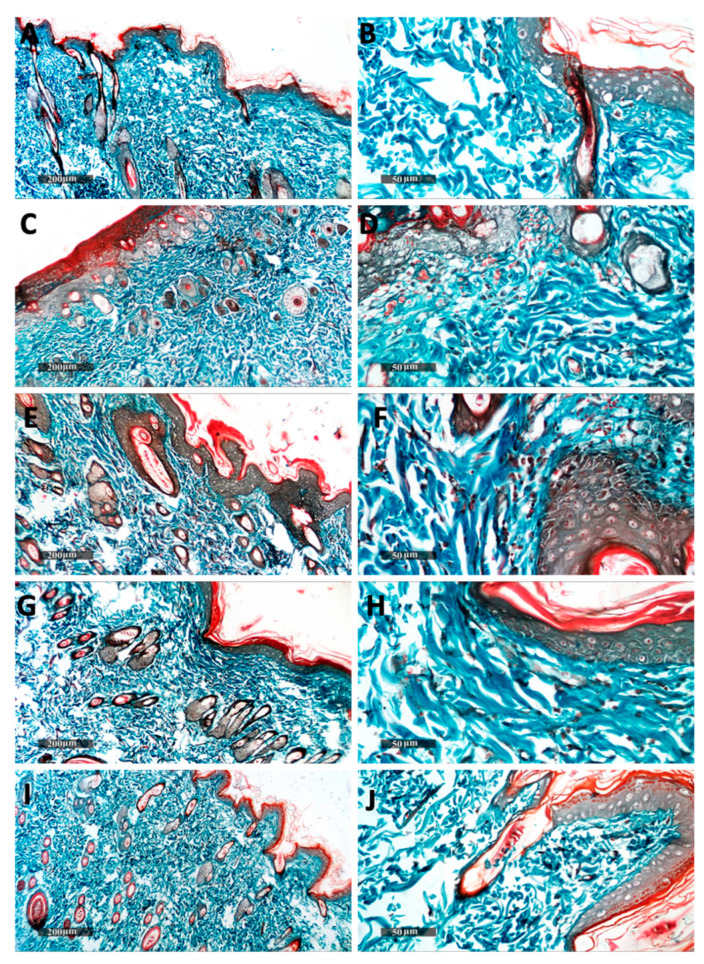
Histopathological photographs showing dermal reactive collagen fibers in different groups; non-UVB-irradiated control group (**A**,**B**). The second group was exposed to UVB for 10 days (**C**,**D**). The remaining groups were all exposed to UV radiation first followed by topical administration of blank BS (1 h before the UVB exposure for 10 days) (**E**,**F**), SPR suspension (1 h before the UVB exposure for 10 days) (**G**,**H**) SPR-BS (1 h before the UVB exposure for 10 days) (**I**,**J**), respectively. Masson’s trichrome stain.

Histopathological evaluation was performed on the skin samples of rats exposed to UVB irradiation. The skin of the normal control group showed no histological alterations with a thin epidermal thickness (15 µm ± 0.45) (Figure 7A,B) and normal dermal collagen fibers (Figure 8A,B). However, acute exposure to UVB produced a significant increase in epidermal thickness up to 5.6-fold compared with a control group. Hyperkeratosis, parakeratosis, and acanthosis were observed in the epidermis. Additionally, epidermal necrosis with inflammatory cells infilteration and fragmented neutrophils was noticed. Intracellular edema in some keratinocytes and karyopyknosis in others were observed. The dermis showed dermatitis characterized by vessel congestion and mononuclear inflammatory cells infilteration (Figure 7C,D). An increase in subepidermal collagen thickness and density was noticed compared with the control group (Figure 8C,D). These histopathological skin alterations come in agreement with the previously reported data mentioned by Acevedo et al. [79] and Abbas et al. [36]. Hyperkeratosis and increased epidermal thickness act as a response for the protection of the dermis from UV damage [80]. The observed dermatitis may have occurred as a result of UVB-induced ROS production stimulating local inflammatory reaction due to pro-inflammatory cytokines release [81]. In contrast, skin from the Blank-BS group showed a slight decrease in epidermal thickness and fewer inflammatory cells infilteration in the dermis with minimal records of vessels congestion (Figure 7E,F) compared with the UVB irradiated group. No changes were found in subepidermal collagen fibers (Figure 8E,F) compared with the UVB-irradiated group. These results are similar to that observed by Abbas et al. [36] who attributed that to the shielding and protective effects of the nanovesicle.

SPR suspension treated group showed moderate protective efficacy with almost intact epidermal layers with evident intact keratinocytes with a significant decrease in epidermal thickness. Moreover, dermal layers showed minimal inflammatory cell infiltration with minimal records of vessel congestion (Figure 7G,H) without change in collagen fibers (Figure 8G,H) compared with the UVB irradiated group. Our results are supported by Jang et al. [82] who found that SPR-derived phycocyanin protects the keratinocytes from UVB-induced damage. This may be attributed to the antioxidative and anti-inflammatory effects of SPR which is confirmed in our biochemical results. SPR-BS treated group showed apparently intact normal organized histological features of skin layers resembling normal control. (Figure 7I,J and Figure 8I,J). Thus, the histopathological findings proved that the SPR-BS is, on a structural basis, providing superior protection for the skin from UVB-induced damage. This could be ascribed to the enhanced anti-inflammatory response of SPR-BS against UVB irradiation which is confirmed in our biochemical results.

**Figure 9:**
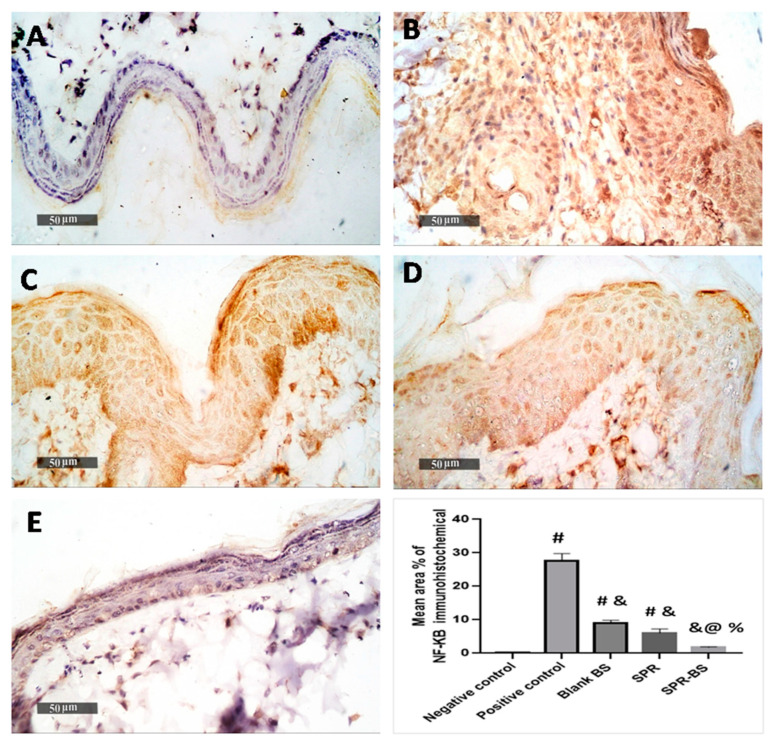
Photomicrographs showing immunohistochemical reactions of p-NFKB in the skin of different groups. (**A**) for non-UV irradiated control group, (**B**) for UVB irradiated group, (**C**) for blank BS group, (**D**) for SPR suspension treated group and (**E**) for SPR-BS group. Area percentage of immunohistochemical expression levels of p-NFKB are expressed as mean ± SD. # Significant from negative control group. & Significant from positive control group. % Significant difference from blank BS. @ Significant from SPR suspension. Significant difference was conducted at *p* < 0.0001.

In immunohistochemical analysis, UVB irradiated group showed a significant increase in p-NFKB immunohistochemical mean expression level (27.5%) compared with the normal control group (0.7%). On the contrary, the blank-BS and SPR groups showed a significant reduction in the mean area percentage of expression value of 9% and 6%, respectively compared with the UVB irradiated group. Furthermore, higher ameliorative efficacy was recorded in the SPR-BL group (1.7% mean expression levels) compared with the UVB-irradiated group (Figure 9). Histological examination and immunohistochemistry results along with ELISA and western blotting analysis demonstrated the superiority of SPR-BS over SPR and blank-BS in preventing skin damage and promoting its healing and regeneration following UV exposure.

#### Limitations

Our study limitation is the use of experimental animals to investigate the effect of our topical preparation. It was mainly conducted to eliminate any concerns regarding irritation from the experimental formulation.

## 3. Materials and Methods

Lipoid S100 (l-α-PC) was bought from Lipoid AG (Ludwigshafen, Germany). Spirulina was a gift from Mega pharma (Alexandria, Egypt). Sodium deoxycholate was bought from Chem-Impex International, Inc. (Wood Dale, IL, USA), and Cholesterol with a molecular weight of 386.654 g/mol was gifted from The Nile for Pharmaceuticals and Chemical Industries (Cairo, Egypt). Other chemicals used in this work were of analytical grade and were obtained from the El-Nasr Company for Pharmaceutical Chemicals (Cairo, Egypt). Catalase (CAT) enzyme-linked immunosorbent assay (ELISA) kit Rat superoxide dismutase (SOD), Matrix metalloproteinase-9 (MMP-9) ELISA kit was purchased from Lifespan Bioscience (Seattle, WA, USA), interleukin 6 (IL-6) was purchased from CUSABIO, Inc. (Wuhan, Hubei, China), Neprilysin ELISA Kit was purchased from Aviscera Bioscience, Santa Clara, CA, USA.

### 3.1. Formulation of Blank and SPR-Loaded BS

Thin-film hydration method was adopted to formulate BS utilizing a rotary evaporator (Heidolph, Germany). Concisely, phosphatidylcholine and cholesterol (4:1) were dissolved in a 10 mL mixture of chloroform and methanol (2:1) in a round bottom flask. The organic phase was gradually vaporized under vacuum at 60 ± 0.5 °C for 15 min using a rotary evaporator (Rotavapor, Heidolph VV 2000, Burladingen, Germany) at 120 rpm. Then, the thin film obtained was rehydrated with 10 mL phosphate buffer saline (pH 7.4, 0.1 M) containing different concentrations of SDC (10% and 25% *w*/*w* of PC) as listed in Table 3. In a bath sonicator, the final mixture was then sonicated at 40 °C till the formation of a milky dispersion [32,33].

Regarding drug loading, in a round bottom flask, SPR was dissolved in the organic solvent–lipid mixture. Then, the previously mentioned steps were followed as aforementioned to obtain SPR-loaded BS. Finally, the dispersion was tightly sealed and stored in a refrigerator overnight (4 °C) for stabilization prior to its characterization [29].

### 3.2. In Vitro Characterization of Blank and SPR-Loaded BS

#### 3.2.1. Particle Size and Zeta Potential Measurements

Particle size (PS), poly dispersity index (PDI), and zeta potential (ZP) were determined for both blank and SPR-loaded bilosomes utilizing the dynamic light scattering (DLS) technique using Zeta sizer Nano ZS. (Malvern, Instruments Ltd., Malvern, UK). All measurements were conducted at 25 °C in triplicates where the results were expressed as mean value ± SD.

#### 3.2.2. Entrapment Efficiency

The % EE of SPR was calculated indirectly by measuring the amount of free SPR in the dispersion medium [83,84]. 1 mL aliquot of SPR BS was centrifuged (Sigma Laboratory Refrigerated Centrifuge, Model 3K-3o, Osterode am Harz, Germany) at 4000 rpm at 4 °C for 1 h using a centrifuge tube fitted with an ultrafilter (Vivaspin 6VR, Sartorius, MWCO 10 kDa, Littleton, MA, USA). The amount of free SPR was measured spectrophotometrically at 620 nm [85]. All measurements were conducted in triplicates. The amount of entrapped SPR was calculated as the difference between the total amount added and the amount in the supernatant using the provided equation [29,86].

$$\%\;EE=\frac{Total\;SPR\;concentration-concentration\;of\;unencapsulated\;SPR}{Total\;SPR\;concentration}\times 100$$

#### 3.2.3. In Vitro Drug Release

In vitro drug release experiment was performed for SPR-BS and free SPR suspension (control) using the bag diffusion technique [29]. Dialysis bags (Visking^®^, MWCO 12,000–14,000, Serva, Denver, CO, USA) containing F3, F4, and SPR were immersed in PBS (pH 6.8) ensuring sink conditions [87]. Bags were placed in a shaking water bath (100 rpm) (Memmert GmbH, Schwabach, Germany) at 32 ± 0.5 °C [29]. At predetermined time intervals, samples were withdrawn and compensated with fresh medium. The amount of SPR released was determined using UV spectroscopy (Shimadzu, model UV-1601 PC, Kyoto, Japan) at 620 nm [85]. The experiment was conducted in triplicates and data were expressed as the mean ± SD. The SPR release mechanism from the developed nanocarrier was investigated using DD Solver 1.0 Software (Microsoft Excel add in program, Microsoft Corporation, Redmond, WA, USA) [88].

#### 3.2.4. Morphological Examination by Transmission Electron Microscopy (TEM)

TEM (JEM-1400; JEOL, Japan) was employed to investigate the morphology of the selected SPR-loaded BS. A saturated solution of uranyl acetate (1% *w*/*v*) was used to stain the samples prior to microscopic examination [45].

#### 3.2.5. Stability during Storage

The stability of the selected BS formulation was assessed after storage at 4 °C for 3 months by examination of particle size, zeta potential, and % EE. All measurements were performed in triplicates.

### 3.3. In Vivo Studies

#### 3.3.1. Animals

The current experiment was approved by the Institutional Animal Ethics Committee of the Faculty of Pharmacy, Damanhour University, Egypt (Ethical Approval Number 722PT33), and complied with the ARRIVE guidelines. The experiments were carried out on the dorsal shaved skin of adult male Wistar rats weighing between 180 and 220 g (6–8 weeks old). The animals were enclosed in plastic cages, kept in an atmosphere maintained at 22 °C ± 3 °C and 50–55% humidity, with a 12-h light–dark cycle, and unrestricted access to water.

#### 3.3.2. Skin Irritation Test

The potential localized reaction between the skin and the selected BS formulation was assessed through the skin irritancy test. The irritancy of SPR suspension, blank BS, and SPR-loaded BS was examined using the Draize method with few amendments [71]. Twenty-four hours preceding the experiment, the rat dorsal side was shaved, and the animals were divided into five groups, 10 rats each, as such:

- Group 1: served as a negative control group.
- Group 2: received 1 mL of the standard irritant, aqueous formalin solution (0.8% *v*/*v*) [89].
- Group 3: received 1 mL of blank BS that was applied once daily for 72 h.
- Group 4: received 1 mL of SPR suspension that was applied once daily for 72 h.
- Group 5: received 1 mL of SPR-BS that was applied once daily for 72 h.

SPR concentration in both SPR suspension and SPR-BS was 1.125% [37].

**Figure 10:**
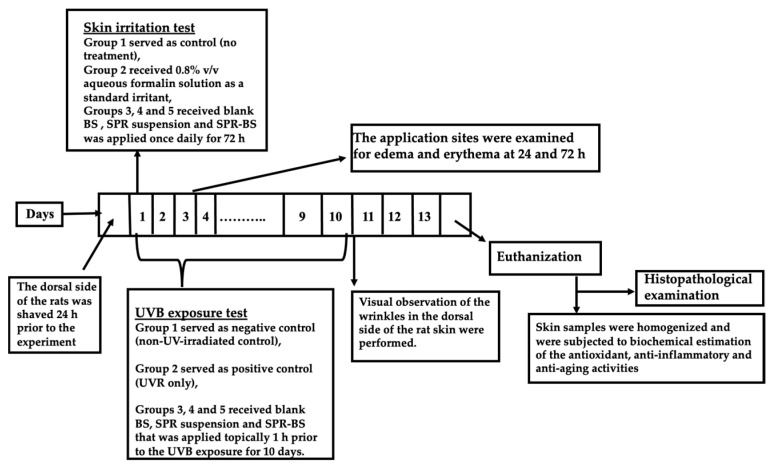
A schematic representation for the experimental design.

After 24 and 72 h, the sites of application were visually monitored for both edema and erythema (Figure 10), and based on a standard score, it was graded from 0 to 4. Scores of 0, 1, 2, 3, and 4 points indicated no edema or erythema; very slight erythema; well-defined erythema; moderate-to-severe erythema; and severe erythema, respectively [63,90]. The final score encompasses the average of 24 and 72 h readings. The primary irritancy index (PII) was deduced for each formula after summing edema and erythema scores; conclusively, the formulations were classified as nonirritant if PII value was <2, irritant if PII value = 2–5, and highly irritant if PII value = 5–8 [71].

#### 3.3.3. UVB Exposure Test

The UVB irradiation system was utilized to stimulate skin photoaging in mice. A UV radiation source with peak emission at 302 nm (CL-1000 M; UVP, Upland, CA, USA) was used as a UVB irradiation source. The UVB irradiation doses were 40–80 mJ/cm^2^, with an exposure time of 15–30 s, and the lamp was fixed 5 cm above the platform where rats were placed [63,91]. Twenty-four hours prior to the experiment, the dorsal side of the rats was shaved. Animals were then divided into five groups (*n* = 10),

- Group 1: Negative control group in which the dorsal skin was shaved while the UV lamp was turned off (non-UV-irradiated control).
- Group 2: Positive control group in which rats were subjected daily for 10 days to UVB irradiation (blank) [89].
- Group 3: Rats received blank BS topically 1 h prior to the UVB exposure for 10 days (UV + blank BS) [63].
- Group 4: Rats received SPR suspension topically 1 h prior to the UVB exposure for 10 days (UV + SPR suspension).
- Group 5: Rats received SPR-BS topically 1 h prior to the UVB exposure for 10 days (UV + SPR-BS).

SPR concentration in both SPR suspension and SPR-BS was 1.125% [37]. At the end of the experiment, about 10 days after the initiation of UVB irradiation, visual observation of the wrinkles in the dorsal side of the rat skin were performed.

#### 3.3.4. Biochemical Analysis by Enzyme Linked Immunosorbent Assay

Biochemical analyses were conducted three days after the final UV irradiation to enable recovery from the effects of acute UV [56]. To assess biochemical parameters, the rats were euthanized by i.p. administration of an overdose of ketamine. Following that, the treated skin was dissected out [63].

To investigate the antioxidant, anti-inflammatory, and antiaging activities of the selected SPR-BS compared to SPR suspension and blank BS, the homogenized skin samples were subjected to biochemical analysis. Several parameters were measured for instance; antioxidant parameters: catalase and SOD; anti-inflammatory markers: IL-6 and TNF α; and antiwrinkling parameters: elastase, MMP 9, and neprilysin.

#### 3.3.5. Western Blotting

Protein samples were extracted from a rat skin to analyze phosphatidylinositol-3-kinase (PI3K) and protein kinase B (AKT) using immunoblotting with a Western blot detection reagent (Amersham Biosciences, Piscataway, NJ, USA) according to the method previously reported by Zhou et al. [92]. The levels of the aforementioned proteins were normalized to that of beta-actin.

#### 3.3.6. Histopathological Study

Skin samples were fixed in 10% neutral buffered formalin for 48 h. Samples were trimmed, then processed, and dehydrated in serial grades of alcohol, cleared in xylene, and then embedded in Paraffin blocks. Five microns tissue sections were cut by microtome then fixed into glass slides and stained with hematoxylin and eosin (H&E) staining for microscopical examination, Masson’s trichrome staining for demonstration of collagen fibers. All standard procedures for sample fixation and staining were done according to Bancroft et al. [93].

#### 3.3.7. Immunohistochemical Staining and Analysis

Immunohistochemical staining was conducted according to the manufacturer’s protocol. Deparaffinized antigen retrieved five microns thick tissue sections and were treated with 3% H_2_O_2_ for 20 min. Washed, incubated with anti p-NFκB p65 antibody (GTX54672, GeneTex Inc., Hsinchu, Taiwan—1:100) 60 min; washed by PBS, followed by incubation with secondary antibody HRP Envision kit (DAKO, Glostrup, Denmark) 20 min; washed by PBS and incubated with diaminobenzidine (DAB) for 10 min. Washed by PBS and then counterstaining with hematoxylin, the tissue section then dehydrated and clearing in xylene then the cover slipped for microscopic examination.

#### 3.3.8. Quantitative Immunohistochemical Analysis

Six random non-overlapping fields from each sample per group were analyzed for obtaining the mean epidermal thickness in H&E staining sections. As well as mean area percentage of positive immunohistochemical expression levels of p-NFκB in immunostained tissue sections. All microscopical examinations, imaging and quantitative analysis using Full HD microscopic imaging system (Leica Microsystems GmbH, Werzlar, Germany) operated by Leica Application software for tissue section analysis.

### 3.4. Statistical Analysis

Statistical analysis of the results was conducted by Prism^®^ 7 (GraphPad Software, Inc., San Diego, CA, USA). For the analysis of colloidal characteristics of bilosomes, a student *t*-test with (*p* < 0.05) was used. The rest of the results were analyzed by applying one-way analysis of variance (ANOVA) followed by Tukey’s test with (*p* < 0.0001).

## 4. Conclusions

In this study, SPR-BS was efficiently developed for the first time as a novel antiaging nanoplatform for the delivery of SPR, a natural bioactive compound. The developed nanodelivery system had acceptable particle size, zeta potential, high entrapment efficiency, and significantly prolonged in vitro drug release. The in vivo antiaging effect was investigated based on the UVB exposure test and immunohistochemical and biochemical analyses quantifying the levels of antioxidant, anti-inflammatory, and antiwrinkling markers. SPR-BS showed superior efficacy. Thus, this novel nanosystem offers better protective antiaging effects than the free drug.

## Figures and Tables

**Table 1:** Mean Particles Size, Polydispersity Index, Zeta Potential and Entrapment Efficiency of blank-BS and SPR-BS. Measurements are expressed as mean ± SD.

Formulation Code	Particle Size (nm)	PDI	Zeta Potential (mV)	Entrapment Efficiency%
F1	241.3 ± 1.87	0.503 ± 0.04	−28.4 ± 1.60	NA
F2	226.5 ± 1.65	0.406 ± 0.05	−33.5 ± 2.03	NA
F3	251.3 ± 1.21	0.310 ± 0.06	−30.6 ± 1.08	85.2 ± 1.50
F4	230.6 ± 1.30	0.305 ± 0.02	−34.5 ± 1.30	89.5 ± 1.08

**Table 2:** Release kinetics of SPR and SPR BS.

Formulation	R^2^			
	Zero Order	First Order	Higuchi	Krosmeyer Peppas
SPR suspension	0.657	0.986	0.484	0.864
F3	0.780	0.977	0.861	0.893
F4	0.776	0.973	0.839	0.886

**Table 3:** Composition of empty and SPR-loaded bilosomes SPR-BS prepared by thin film hydration method.

Ingredients	F1	F2	F3	F4
Phosphatidyl choline (PC) mg %*w*/*v*	100	100	100	100
Cholesterol (Chol) mg %*w*/*v*	25	25	25	25
Sodium deoxycholate (SDC) mg %*w*/*v*	10	25	10	25
Spirulina (SPR) mg %*w*/*v*	--------	--------	100	100

## Data Availability

Not applicable.

## Supplementary Materials

The following supporting information can be downloaded at: https://www.mdpi.com/article/10.3390/ph16010036/s1, Figure S1: Particle size distribution of different formulations. Figure S2: Zeta potential distribution of different formulations. Figure S3: (A) SPR UV spectrum and (B) SPR calibration curve in PBS pH 7.4.
