# Supplementary material for: Novel Siprulina platensis Bilosomes for Combating UVB Induced Skin Damage
Source: Pharmaceuticals (Basel). 2022 Dec 27;16(1):36. doi: 10.3390/ph16010036 (PMC9865528; doi:10.3390/ph16010036)
Supplement: Supplementary file 1 [file pharmaceuticals-16-00036-s001.zip › pharmaceuticals-2095412-supplementary.pdf]

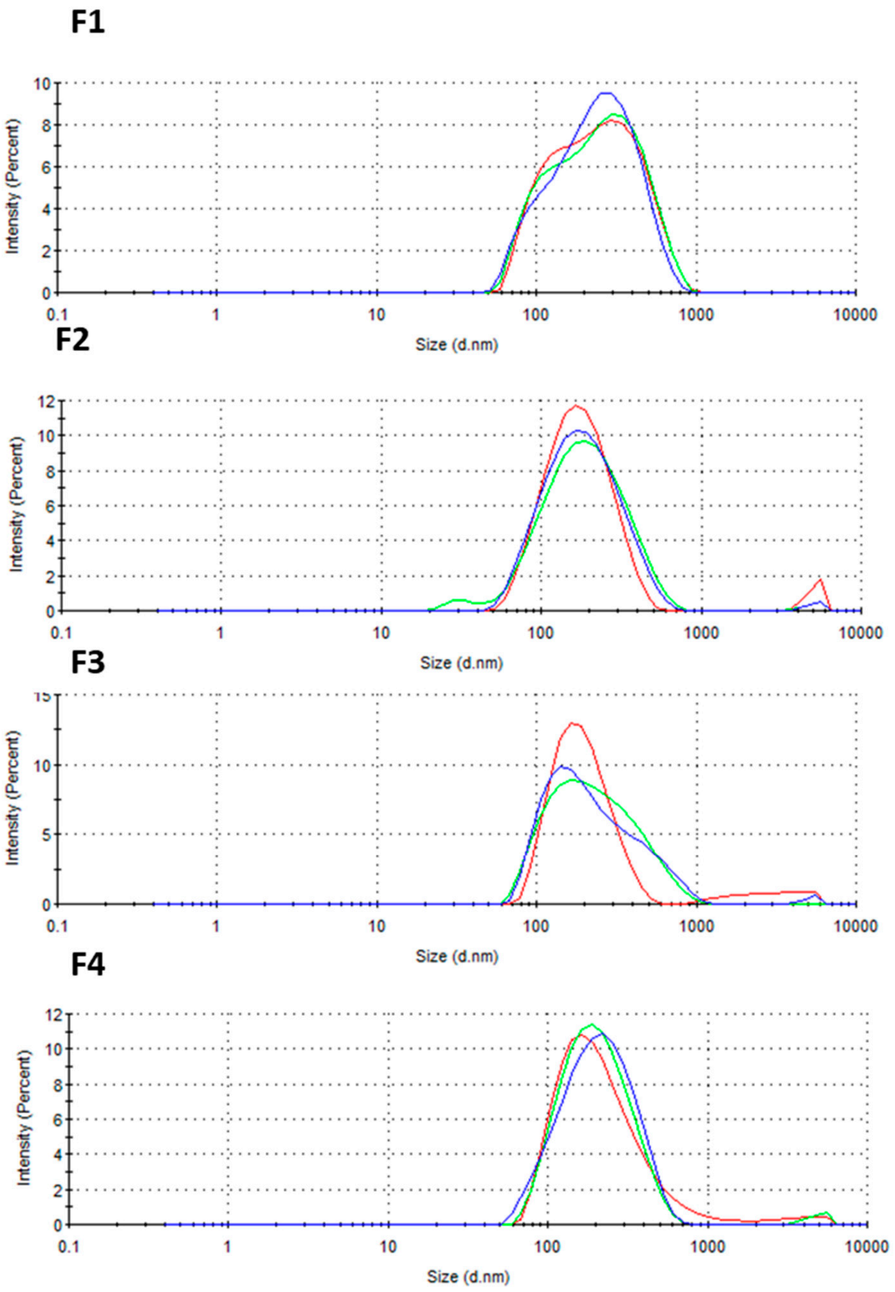

Figure S1: Particle size distribution of different formulations.

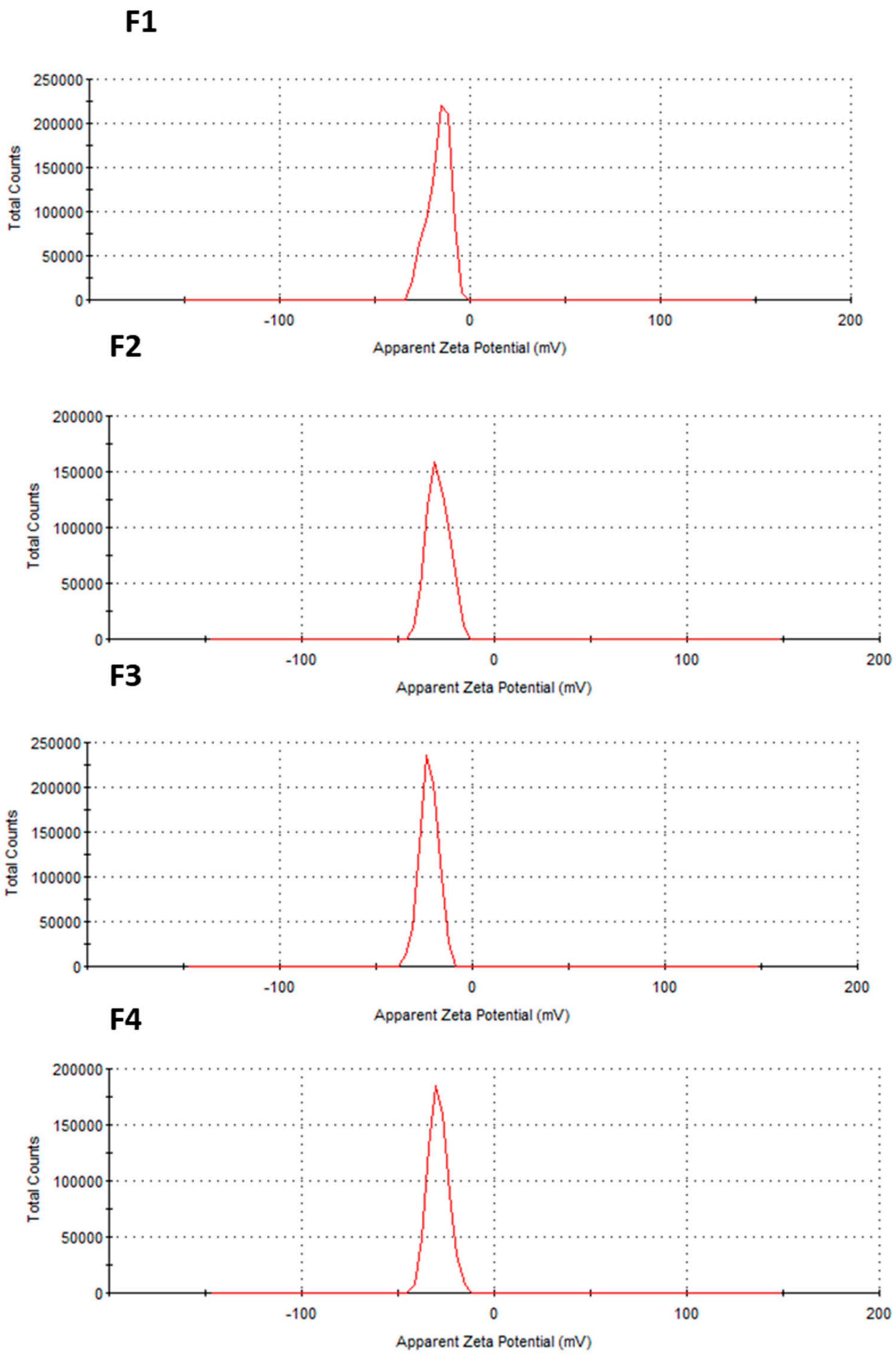

Figure S2: Zeta potential distribution of different formulations.

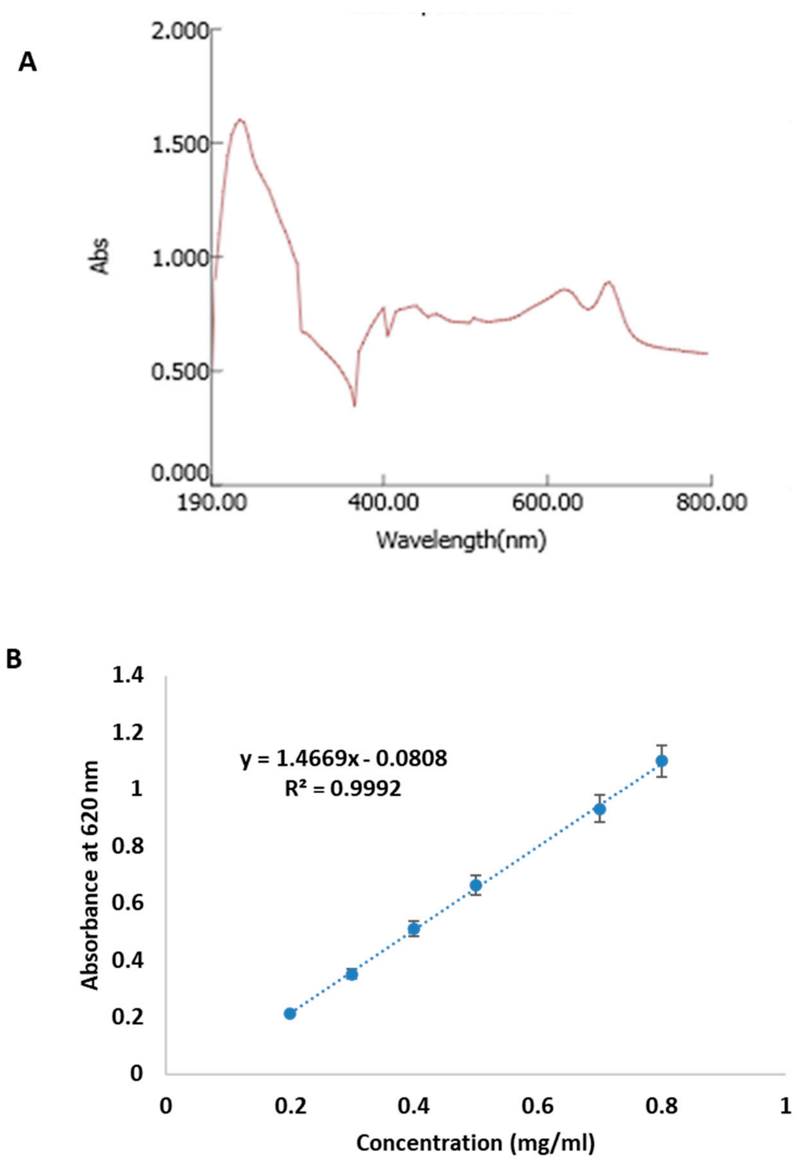

Figure S3: (A) SPR UV spectrum and (B) SPR calibration curve in PBS pH 7.4.
